# Quantitative Risk Assessment of Wind-Supported Transmission of Highly Pathogenic Avian Influenza Virus to Dutch Poultry Farms via Fecal Particles from Infected Wild Birds in the Environment

**DOI:** 10.3390/pathogens13070571

**Published:** 2024-07-08

**Authors:** Clazien J. de Vos, Armin R. W. Elbers

**Affiliations:** Department of Epidemiology, Bioinformatics &Animal Models, Wageningen Bioveterinary Research, P.O. Box 65, 8200 AB Lelystad, The Netherlands; armin.elbers@wur.nl

**Keywords:** HPAI, wild birds, QMRA, fecal droppings, aerosolization, wind

## Abstract

A quantitative microbial risk assessment model was developed to estimate the probability that the aerosolization of fecal droppings from wild birds in the vicinity of poultry farms would result in the infection of indoor-housed poultry with highly pathogenic avian influenza virus (HPAIv) in the Netherlands. Model input parameters were sourced from the scientific literature and experimental data. The availability of data was diverse across input parameters, and especially parameters on the aerosolization of fecal droppings, survival of HPAIv and dispersal of aerosols were uncertain. Model results indicated that the daily probability of infection of a single poultry farm is very low, with a median value of 7.5 × 10^−9^. Accounting for the total number of poultry farms and the length of the bird-flu season, the median overall probability of at least one HPAIv-infected poultry farm during the bird-flu season is 2.2 × 10^−3^ (approximately once every 455 years). This is an overall estimate, averaged over different farm types, virus strains and wild bird species, and results indicate that uncertainty is relatively high. Based on these model results, we conclude that it is unlikely that this introduction route plays an important role in the occurrence of HPAIv outbreaks in indoor-housed poultry.

## 1. Introduction

Wild water birds of the orders Anseriformes and Charadriiformes are the natural reservoir of avian influenza virus [1]. Wild water birds can play an important role in directly infecting poultry in free-range operations or bringing the virus to the environment in close vicinity to poultry units [2]. Avian influenza viruses are categorized as low-pathogenic avian influenza virus (LPAIv) or highly pathogenic avian influenza virus (HPAIv), based on the pathogenicity of the virus in chickens and the amino acid sequence of the connecting peptide of the haemagglutinin molecule (HA0) (i.e., the cleavage site) [3]. In general, LPAIv infections may be asymptomatic and produce no or mild disease in chickens [4], while HPAIv infections produce high morbidity and mortality in poultry [5].

Genetic comparisons of HPAIv strains from outbreaks on Dutch poultry farms since 2014 and HPAIv strains isolated from dead wild birds strongly suggest that these HPAIv strains were carried to the Netherlands by migratory wild birds from Asia, possibly through overlapping flyways and common breeding sites in Siberia [6,7,8]. Velkers et al. [9] showed a marked increase in birds of the Anatidae family around poultry farms occurring from October to April in the Netherlands. This increase was more pronounced for farms close to wetlands compared to farms in other areas. The most striking increase was found for the Eurasian wigeon (*Anas penelope*), with bird densities several tens of folds higher around farms located close to low-lying wetlands. Eurasian wigeons were one of the predominant species with massive mortality due to HPAIv in 2016–2017 and were also found in the vicinity of poultry farms with phylogenetically related H5N8 virus outbreaks [7]. Therefore, the indoor housing of all free-range poultry was made mandatory during the bird-flu season. Despite the fact that indoor housing prevents direct contact between poultry and infected wild avian species, many poultry farms still became infected.

HPAIv multiplies in the respiratory, intestinal, renal and reproduction organs of infected birds. Infected birds excrete the virus via secreta (fluid secretion) from the nose and mouth/beak, conjunctiva (mucous membrane of the eyes) and excreta (feces) from the cloaca [10]. When infected wild birds excrete the virus, they can infect poultry via either direct or indirect contact. Indirect transmission of the virus between wild birds and poultry can occur if, e.g., the environment or fomites are contaminated with HPAIv in secretions and excreta from infected wild birds. An example of an indirect transmission pathway would be via contaminated water (e.g., a pond or puddles of water) or contaminated soil in the free-range area of a poultry farm. Wild birds regularly visit the outdoor facilities of commercial poultry farms and can therefore contaminate the free-range area with HPAIv via secretions and excreta, such as fecal droppings [2,11]. Another indirect transmission pathway would be materials, shoes, clothing, stable equipment, vehicles, etc. that are contaminated with secretions and excreta from HPAI-infected wild birds in the area outside a poultry unit and brought into the poultry house by the farmer and his/her family members or professional visitors like consultants, veterinary practitioners, catchers and vaccination crews [12]. The airborne transmission of HPAIv is another indirect transmission route and is considered a possible transmission route between nearby poultry farms during epidemics with large numbers of infected poultry farms [13,14,15]. HPAIv-infected poultry can produce large quantities of virus that, when stuck to particulate matter (PM), such as aerosolized dust originating from feces, bedding material, feed and feathers, can become airborne [16]. This airborne virus can be transported by forced ventilation air from a house with infected poultry to the environment outside, which could lead to the potential wind-borne spread of the virus to other poultry farms [17,18]. However, a recent study by James et al. [19] showed that airborne particles harboring infectious HPAIv originating from poultry houses with HPAIv-infected poultry can be translocated only over short distances (<10 m) through the air, while macroscopic particles containing viral RNA (non-infectious) might travel further (≤80 m). They concluded that the potential for the airborne transmission of clade 2.3.4.4b H5N1 HPAIv between poultry farms is considered low.

One of the hypotheses suggested by poultry farmers to explain HPAIv outbreaks in poultry farms with indoor-housed animals is the wind-supported transport of particles from fecal droppings from HPAIv-infected wild birds in the surroundings of the farm via the air inlets of the poultry house, resulting in the exposure of the poultry inside the house [20]. Therefore, the aim of our study was to build a quantitative microbial risk assessment (QMRA) model to estimate the probability that this indirect transmission route would indeed result in the infection of indoor-housed domestic poultry.

## 2. Materials and Methods

**Figure 1:**
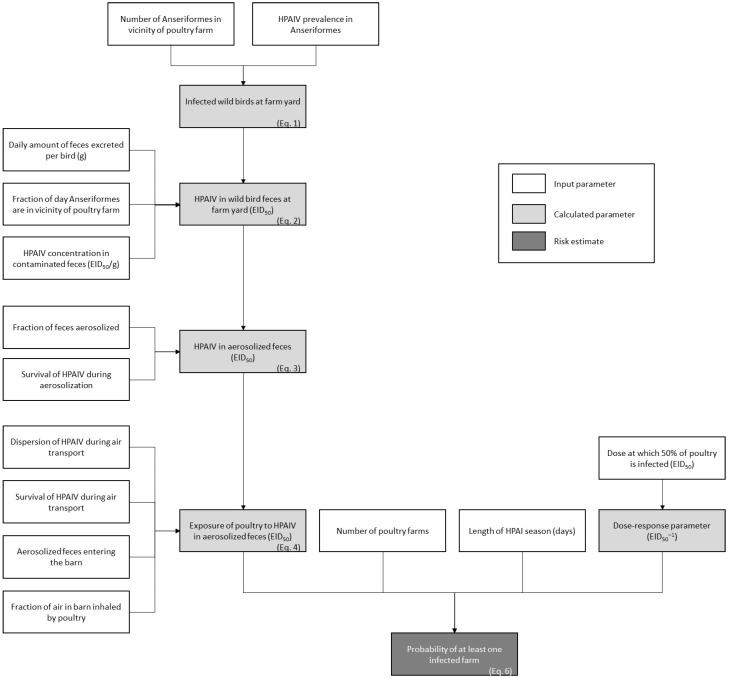
Outline of the quantitative microbial risk assessment model to estimate the HPAI transmission risk from wild birds to domestic poultry via aerosolized fecal droppings.

A QMRA model was built to estimate the probability that the aerosolization of fecal droppings of wild birds would result in infection of indoor-housed domestic poultry. The main conditions for this exposure route are the presence of infected wild birds close to the poultry house, the excretion of infectious virus in feces, the subsequent aerosolization of fecal droppings and the survival of infectious virus during aerosolization and air transport (Figure 1). To characterize the risk, the estimated exposure of poultry to HPAIv was combined with the bird infectious dose at which 50% of exposed poultry is expected to be infected (BID50) in an exponential dose–response model.

The QMRA model is a stochastic risk model built in Microsoft Excel for Microsoft 365 MSO (Version 2308 Build 16.0.16731.20542) and @Risk 8.3.2 (Lumivero, Denver, CO, USA) [21]. Model input parameters were sourced from the scientific literature and experimental data. The availability of data was diverse across input parameters, and especially input parameters on the aerosolization of feces, survival of the virus and dispersal of aerosols were quite uncertain. Probability distributions were used to represent uncertainty on input parameters. The model does not, however, account for variability across poultry farms, wild bird species or HPAIv strains. We challenged some of our assumptions and input parameters in a what-if analysis.

We estimated the transmission risk from wild birds to domestic poultry via aerosolized fecal droppings under the conditions of a “normal” Dutch bird-flu season, which is approximately from October to April. We considered birds of the order Anseriformes to pose the highest risk to farms, because of both the relatively high prevalence of HPAI viruses in this order [22,23,24] and their abundant presence in the environment around some poultry farms during the bird-flu season [9].

The output of the model is the probability that the exposure of indoor-housed domestic poultry to aerosolized fecal droppings from wild birds will result in at least one infected poultry farm during the bird-flu season. The model was run for 10,000 iterations, and results are given as median values and 95% uncertainty intervals.

### 2.1. Model Calculations

The model consists of two steps: first, the exposure of poultry to HPAIv via aerosolized fecal droppings of wild birds is assessed, after which the exposure is combined with a dose–response model to estimate the daily probability of infection of a single poultry farm. Results are then combined with the number of poultry farms in the Netherlands and the length of the bird-flu season (in days) to estimate the overall probability of HPAI introductions to poultry farms during the Dutch bird-flu season via this exposure route.

#### 2.1.1. Exposure Assessment

The number of infected Anseriformes visiting a poultry farm (${WB}_{inf}$) was calculated as:

(1) $${WB}_{inf}=WB\times {Prev}_{wb}$$

where WB is the number of wild birds approaching to the farm, and ${Prev}_{wb}$ is the apparent prevalence of HPAI in wild birds.

To estimate the daily amount of HPAIv (EID_50_) present in wild bird feces on the farm yard (${AI}_{fec,d}$), we multiplied ${WB}_{inf}$ with the daily amount of feces excreted by wild birds (gram wet feces) (${Fec}_{wb,d}$), the fraction of the day the wild birds stay (and excrete) in close vicinity to the farm ($F_{pf}$) and the average concentration of HPAIv (EID_50_/gram) in feces of wild birds (${AI}_{fec\_conc}$) as follows:

(2) $${AI}_{fec,d}={WB}_{inf}\times {Fec}_{wb,d}\times F_{pf}\times {AI}_{fec\_conc}$$

To estimate the amount of HPAIv that is released into the air via aerosolization (${AI}_{air,d}$), we multiplied ${AI}_{fec,d}$ with the probability that the weather conditions during the bird-flu season are favorable for drying the feces ($F_{weather}$) and the expected survival of HPAIv during drying of feces ($F_{surv\_dry}$) as follows:

(3) $${AI}_{air,d}={AI}_{fec,d}\times F_{weather}\times F_{surv\_dry}$$

The amount of aerosolized HPAIv that poultry in the poultry house is then exposed to (${AI}_{exp,d}$) was calculated as the product of ${AI}_{air,d}$ and the fraction of virus reaching the animals, accounting for the retained fraction of virus after the dispersion of the aerosols over a short distance ($F_{disp}$), as well as the survival of the virus during air transport ($F_{surv\_trans}$), the fraction of aerosols entering the barn ($F_{barn}$) and the fraction of air in the poultry house that is inhaled by the animals ($F_{inhale}$):

(4) $${AI}_{exp,d}={AI}_{air,d}\times F_{disp}\times F_{surv\_trans}\times F_{barn}\times F_{inhale}$$

#### 2.1.2. Risk Characterization

To estimate the daily probability of at least one infected bird in the poultry farm ($P_{inf,pf,d}$), an exponential dose–response model was used:

(5) $$P_{inf,pf,d}=1-{exp}^{-\left(DR\times {AI}_{exp,d}\right)}$$

where DR is the exponential dose–response parameter.

The overall probability of at least one infected poultry farm during the bird-flu season ($P_{inf}$) was estimated by including the number of poultry farms in the Netherlands ($N_{pf}$) and the length of the bird-flu season in days (D) in the dose–response model as follows:

(6) $$P_{inf}=1-{exp}^{-\left(DR\times {AI}_{exp,d}\times N_{pf}\times D\right)}$$

### 2.2. Model Input

#### 2.2.1. Exposure Assessment

The number of Anseriformes in close vicinity to a poultry farm (WB) is likely to be low. Since we assumed that only bird droppings on paved surfaces could dry enough and subsequently aerosolize during winter time [25], an estimate was made of the expected number of birds in a radius of <20 m of the poultry house. Few studies have actually closely monitored wild bird activity on poultry farms [2,20,26,27,28,29,30]. To estimate the number of Anseriformes visiting poultry farms and the time spent on the farm yard, we used observations by Elbers and Gonzales [2] on the daily number of mallards visiting the free-range area of a layer farm in a high-risk area in the Netherlands during the bird-flu season. Observed numbers of mallards varied widely across months (with a peak from December to February) and days, with mallards observed on 42 out of 136 days. The median number of birds observed on these days was 6 (95% uncertainty interval: 1 to 24). Mallards mostly visited the farm during night time and spent a median of 3.7 h per day in the free-range area (95% uncertainty interval: 0.04 to 18.4). These latter values were used to model the fraction of the day in which birds stay in close vicinity to the farm ($F_{pf}$) (Table 1).

The probability that a wild bird visiting the poultry farm would be infected with HPAIv was based on field studies in the Netherlands during the bird-flu seasons of 2014/15 and 2016/17, in which live birds were captured and sampled using oropharyngeal and cloacal swabs, and fecal samples were collected in the field [22,23,24]. Only samples from Anseriformes (ducks and geese) were used to estimate the apparent prevalence (${Prev}_{wb}$). A total of 8554 samples were tested, of which 34 were positive for HPAI, resulting in an apparent prevalence of 4.1 × 10^−3^. Uncertainty in the estimate of ${Prev}_{wb}$ was simulated using a beta distribution. More recent data from the United Kingdom (UK) (2019/20) and Italy (2020/21) report higher apparent prevalences in wild waterfowl ranging from 1.4 × 10^−2^ to 4.6 × 10^−2^ [41,42]. This is in agreement with higher numbers of reported HPAI cases in both wild birds and poultry in recent years [43,44,45]. We challenged our model assumptions using the data from these studies to estimate the HPAI prevalence in wild birds in the what-if analysis (scenario WI-1).

The daily amount of feces excreted by wild birds (${Fec}_{wb,d}$) was based on data for adult mallards (*Anas platyrhynchos*), with an average value of 36.9 g dry weight per bird per day [31]. Only adult mallards were considered, as nestlings are not likely to be present during the bird-flu season. The moisture content of duck droppings varies between 70 and 90% [25,32,46,47]. To obtain wet weight values, dry weights were therefore multiplied by a factor of 5 [32]. Gere and Andrikovics [48] reported lower dry weights of fecal droppings from mallards (26.3 g per day), and we evaluated the impact of changing this parameter in the what-if analysis (scenario WI-2). A second what-if scenario (WI-3) was run to mimic the risk of geese droppings, which have a higher dry weight. We used the reported fecal dry weight of the greylag goose (*Anser anser*) (100 g per day) [32], which is an abundant species in the Netherlands during the bird-flu season [49].

The concentration of HPAIv in the feces of wild birds (${AI}_{fec\_conc}$) was derived from the results of a systematic literature review by Germeraad et al. [11], with an estimated mean of 3.8 log_10_ EID_50_/mL. This estimate is close to values used in other modeling studies (e.g., [38]). However, more recent experiments in Pekin ducks (*Anas platyrhynchos domesticus*) and Eurasian wigeons (*Anas penelope*) indicated a high difference in shedding levels of the HPAI H5 viruses present in 2014 (group A virus) compared to those present in 2016 and 2017 (group B viruses) [50]. The estimates from these experiments were used in the what-if analysis. Scenario WI-4 is based on excretion data from Eurasian wigeons for H5N8-2014 with an estimated mean of 3.4 log_10_ EID_50_/mL, whereas scenario WI-5 is based on excretion data from the same bird species for H5N8-2016 with an estimated mean of 5.0 log_10_ EID_50_/mL.

The probability that feces are aerosolized is highly dependent on the weather conditions. Elbers [25] observed the drying of feces only when deposited on a concrete surface (not on grassland) on days with no precipitation and global sun irradiation ≥ 1000 J/cm^2^. To estimate the probability that weather conditions during the bird-flu season are favorable for drying of feces to allow for HPAIv release into the air ($F_{weather}$), we determined the number of days from October to April in a 30-year period (1993–2022) that had suitable weather conditions lasting for at least 7 days [33]. This resulted in an estimated 1.4% of days allowing for the aerosolization of feces (Supplementary Table S1). This estimate is likely to result in an overestimate of the risk, as a single week of drying is probably not sufficient for full aerosolization. Elbers [25] measured 23% remaining moisture in duck feces after a week of suitable weather conditions. On the other hand, we might have underestimated the risk using this value considering that feces excreted during previous days are also subject to aerosolization once the suitable weather period starts and that HPAIv can survive for prolonged periods (>30 days) at low ambient temperatures [51,52].

The drying of feces results in the quick inactivation of HPAIv; Zarkov and Urumova [34] recorded a 3.25 log_10_ inactivation of LPAIV (H6N2) after one day of drying, and no virus was detected after two days of drying. This is in accordance with observations by Shortridge et al. [51] that H5N1 virus was inactivated within one day when dried at a temperature of 25 °C. Sedlmaier et al. [53] spiked a suspension of dried broiler manure with LPAI virus (H10N7), after which they nebulized the suspension and measured the virus concentration in deposited fecal PM_2.5_ (particle size < 2.5 mm) on filters. They did retrieve viable virus in the deposited PM_2.5_, but it is not clear how these virus concentrations relate to the original concentrations in the broiler manure suspension. As we expect a minimum drying period of seven days to allow for the aerosolization of fecal droppings during the Dutch bird-flu season [25], it is not very likely that viable virus is still present in fecal dust particles. As a worst-case assumption, we used the 3.25 log_10_ reduction given by Zarkov and Urumova [34] to quantify the expected survival of HPAIv during the drying of feces ($F_{surv\_dry}$) in the baseline model calculations. In the what-if analysis (scenario WI-6), we assumed that virus reduction due to drying was twice as high (6.5 log_10_).

Survival of virus during air transport ($F_{surv\_trans}$) is highly dependent on the environmental conditions and the time from aerosolization to the exposure of poultry. At low temperatures, Harper [36] reports survival rates of influenza in aerosols from 61% to 70% after 1 h and 3% to 19% after 23 h, depending on relative humidity. In the model calculations, we used the values for 1 h survival, as we assumed the fecal aerosols will be released close to the poultry houses and will not need much time to reach the barn. In the what-if analysis (scenario WI-7), we used the decay rate constant given by Ssematimba et al. [17] and estimated the surviving virus fraction after 1 h at 99%.

We also accounted for the loss of infectivity due to the dispersal of the virus ($F_{disp}$) using model results given by Lighthart and Mohr [35], indicating a 0.5 log_10_ dilution at 10 m distance and a 2 log_10_ dilution at 20 m distance. We acknowledge that this value will vary depending on weather conditions (wind, humidity). In the what-if analysis (scenario WI-8), we used results from dispersion calculations from Sedlmaier et al. [53] for particulate matter (PM_10_) downwind from a broiler farm. Based on these proxy data, the expected decrease in concentration of the virus after traveling a distance of 10 to 20 m is between 63% and 78%.

Furthermore, the aerosols will not always reach the poultry house and enter the barn via the ventilation openings. We assumed that the fraction of aerosols entering the barn ($F_{barn}$) will on average be 5% based on the relative surface area of ventilation openings in the side walls of both the layer farm and the broiler farm included in the study described by Elbers et al. [20]. This might be an underestimate, as underpressure in the barn resulting from a negative-pressure ventilation system will result in a slightly higher fraction of aerosols entering. On the other hand, the estimate of $F_{barn}$ is an overestimate, as we did not account for the fact that the wind direction will not always favor dispersion of aerosols to the barn. As both effects could not be quantified, we used the 5% value as a worst-case estimate, assuming that aerosols will be directed to the poultry house most of the times.

The fraction of air inhaled by the animals in the poultry house ($F_{inhale}$) was estimated for laying hens and broilers separately using data on the ventilation rate of poultry houses and the respiratory rate of chickens (Table 1). The ventilation rate determined the time span during which the aerosols would be present in the barn, and this time span combined with the respiratory rate determined the fraction of aerosols the animals could maximally inhale. In the baseline scenario, values for laying hens were used, as laying hens are most at risk for HPAIv introduction [54,55]. In the what-if analysis (scenario WI-9), values for broilers were used.

#### 2.2.2. Risk Characterization

The exponential dose–response parameter was derived from the infectious dose that yields 50% probability of infection in birds, the bird infectious dose ${BID}_{50}$. This dose is dependent on the poultry species, the virus strain and the inoculation route [25]. The reported bird infectious doses of HPAIv in ducks are lower than those in chickens [56,57,58]. Swayne and Slemons [59] estimated the bird infectious dose of HPAIv in chickens to vary between 1.2 and 4.7 log_10_ EID_50_, with values for H5N1 strains from Asian outbreaks since 1997 between 2.3 and 3.1 log_10_ EID_50_. Pantin-Jackwood et al. [57] estimated slightly higher ${BID}_{50}$ values for more recent H5N1 and H5N8 strains, varying between 2.6 and 4.2 log_10_ EID_50._ These ${BID}_{50}$ values were all based on intranasal inoculation. Birds are, however, approximately 30 times more sensitive to the aerosol route of infection [39], which implies that ${BID}_{50}$ values for aerosol exposure are lower than those for intranasal inoculation. Estimated values of the ${BID}_{50}$ for the aerosol route indeed indicate higher sensitivity, with most of them estimated at approximately 1 log_10_ EID_50_ [39]. There is no evidence that HPAIv strains derived from other poultry species, such as ducks, result in a significantly higher ${BID}_{50}$ in chickens [25,39,59]. In the baseline model calculations, we used a ${BID}_{50}$ with a mean value of 1.2 log_10_ EID_50_, based on observations by Sergeev et al. [39]. In the what-if analysis (WI-10), we used a much higher value of 4.9 log_10_ EID_50_ based on observations of the ${BID}_{50}$ of H5N8 virus isolated from a tufted duck (*Aythya fuligula*) [25,60].

To estimate the overall probability of at least one infected poultry farm during the Dutch bird-flu season ($P_{inf}$), the exposure of a single farm on a single day was multiplied by the total number of poultry farms in the Netherlands and the length of the bird-flu season (212 days). In 2022, the number of laying farms in the Netherlands was 734, and the number of broiler farms was 619 [40].

### 2.3. Uncertainty Analysis

To evaluate the sensitivity of model results for uncertain input parameters, correlation coefficients between sampled values of these parameters and model results for the overall probability of at least one infected poultry farm during the bird-flu season ($P_{inf}$) were calculated. Furthermore, what-if scenarios were run with the model to evaluate the impact of modeling assumptions (see also Section 2.2). An overview of the what-if scenarios is given in Table 2.

## 3. Results

### 3.1. Baseline Scenario

**Figure 2:**
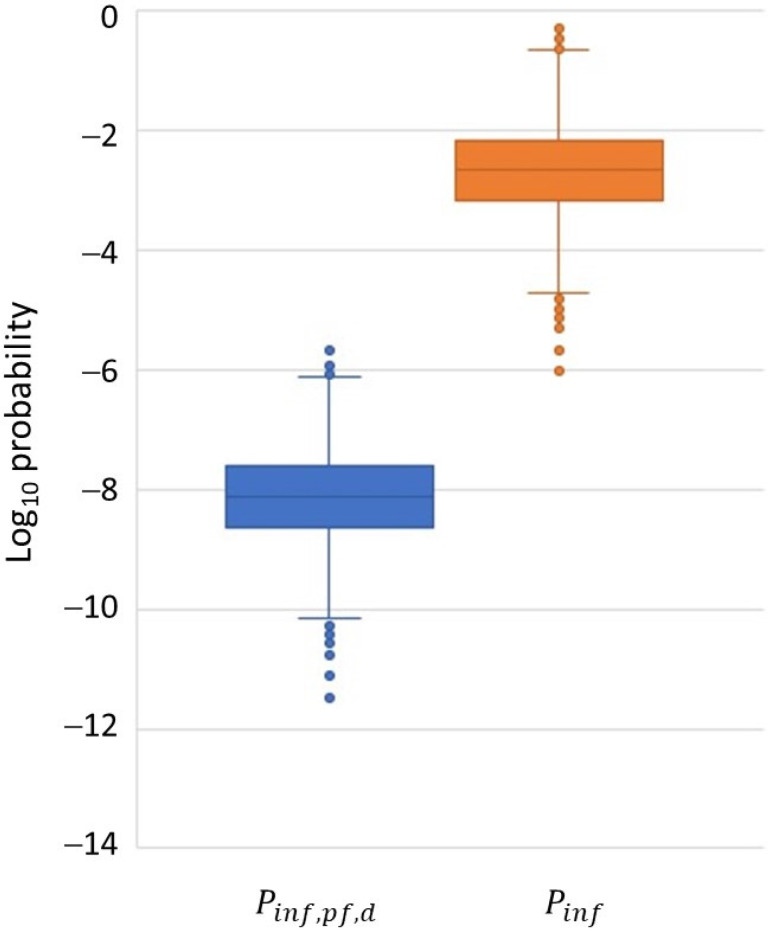
Box-and-whisker plot of model results for the daily probability of infection of a single poultry farm ($P_{inf,pf,d}$) and the overall probability of at least one infected poultry farm during the bird-flu season ($P_{inf}$).

The estimated daily probability of the infection of a single poultry farm ($P_{inf,pf,d}$), i.e., at least one infected bird present in the farm, is very low, with a median value of 7.5 × 10^−9^ (95% uncertainty interval: 2.5 × 10^−10^ to 2.0 × 10^−7^). The box-and-whisker plot in Figure 2 provides more insight into the uncertainty distribution of results. When accounting for the total number of poultry farms in the Netherlands and the length of the bird-flu season, this results in a median overall probability of at least one infected poultry farm during the bird-flu season ($P_{inf}$) of 2.2 × 10^−3^ (95% uncertainty interval: 7.1 × 10^−5^ to 0.06). In other words, an HPAI outbreak in a poultry house due to the wind-supported transmission of HPAIv via fecal particles from infected wild birds is expected to happen approximately once every 455 years. The median daily exposure of poultry to HPAIv in aerosols on a single farm (${AI}_{exp,d}$) is 1.7 × 10^−7^ EID_50_ (95% uncertainty interval: 6.1 × 10^−9^ to 3.9 × 10^−6^). It should be noted that this is an averaged value over all days, i.e., days with and without infected wild birds visiting the farm yard and days with and without weather conditions suitable for the aerosolization of wild bird droppings. The probability that infected wild bird droppings are present and conditions for aerosolization are met on a single day is 1.3 × 10^−4^ (95% uncertainty interval: 3.3 × 10^−5^ to 3.3 × 10^−4^). On these days favorable for transmission, the estimated median exposure is 1.4 × 10^−3^ EID_50_ (95% uncertainty interval: 1.1 × 10^−4^ to 3.2 × 10^−2^), resulting in an infection probability of 5.9 × 10^−5^ (95% uncertainty interval: 5.5 × 10^−6^ to 1.8 × 10^−3^) for individual poultry farms.

### 3.2. Uncertainty Analysis

#### 3.2.1. Sensitivity Analysis

**Figure 3:**
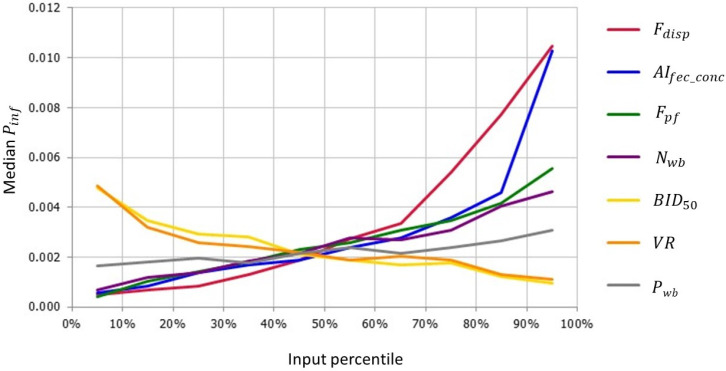
Spider plot showing the relation between the median overall probability of at least one infected poultry farm during the bird-flu season ($P_{inf}$) and the percentile values of input parameters that had a correlation coefficient > |0.1| with $P_{inf}$. These input parameters were: fraction of virus retained after the dispersion of aerosols over a short distance ($F_{disp}$); concentration of HPAIv in wild bird feces (${AI}_{fec\_conc}$); fraction of the day that wild birds spent at the farm yard ($F_{pf}$); number of wild birds at the farm yard on a day that birds are present ($N_{wb}$); bird infectious dose (${BID}_{50}$); ventilation rate of poultry house (VR); and daily probability that wild birds are present at the farm ($P_{wb}$).

Ten parameters of the model were inputted as uncertainty distributions (Table 1). Seven of these uncertain model input parameters had a correlation coefficient ≥ |0.1| for the overall probability of at least one infected poultry farm during the bird-flu season ($P_{inf}$) (Supplementary Table S2), indicating that results of the model are sensitive to uncertainties in these parameters. Figure 3 shows the change in the median $P_{inf}$ when these input parameters were changed from their lowest to highest value (input percentile values at x-axis). Uncertainty in the retained fraction of the virus after the dispersion of aerosols over a short distance ($F_{disp}$) and the concentration of HPAIv in the feces of wild birds (${AI}_{fec\_conc}$) had the greatest impacts on model results. Taking the 95th percentile values of these input parameters resulted in a 4.7- to 4.8-fold increase in $P_{inf}$. The bird infectious dose (${BID}_{50}$) and the ventilation rate of poultry houses (VR) were negatively correlated with $P_{inf}$. Taking the 5th percentile values of these input parameters resulted in a 2.2-fold increase in $P_{inf}$.

#### 3.2.2. What-If Analysis

**Figure 4:**
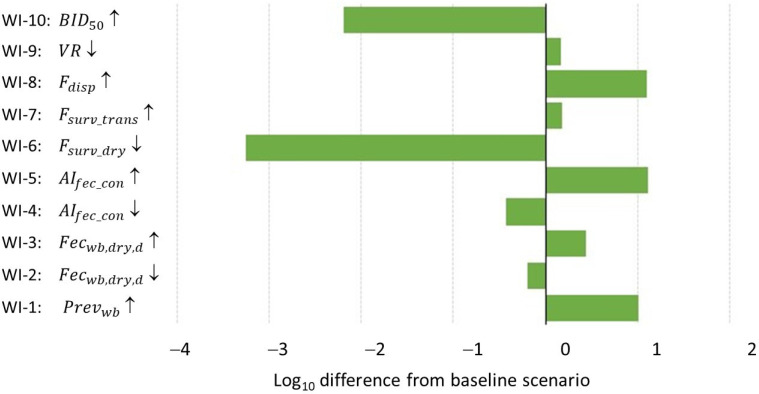
Tornado chart showing the relative increase or decrease (expressed as log_10_ difference) in the overall probability of at least one infected poultry farm during the bird-flu season ($P_{inf}$) compared to the baseline scenario for 10 what-if scenarios. Parameters considered in the what-if scenarios were: apparent HPAI prevalence in wild birds (${Prev}_{wb}$); daily amount of feces excreted by wild birds (${Fec}_{wb\_dry,d}$); concentration of HPAIv in wild bird feces (${AI}_{fec\_conc}$); survival of HPAIv during the drying of feces ($F_{surv\_dry}$); survival of HPAIv during air transport ($F_{surv\_trans}$); fraction of virus retained after the dispersion of aerosols over a short distance ($F_{disp}$); ventilation rate of poultry house (VR); and bird infectious dose (${BID}_{50}$). The arrows indicate an increase (↑) or a decrease (↓) of the input parameter’s value. A more detailed description of each scenario is given in Table 2.

Results of the what-if analysis are given in Figure 4. Three what-if scenarios resulted in a 10-fold increase in the risk compared to the baseline scenario: a higher HPAI prevalence in wild birds based on more recent studies in wild birds in the United Kingdom and Italy (WI-1), a higher concentration of HPAIv in feces based on data for H5N8-2016 (WI-5) and a higher fraction of infected aerosols retained during the dispersion of aerosols (WI-8). Two what-if scenarios resulted in a significant decrease in the risk compared to the baseline scenario: a lower probability of the survival of HPAIv during aerosolization (WI-6) and a higher bird infectious dose based on H5N8 virus isolated from a tufted duck (WI-10). The amount of feces excreted by wild birds (WI-2 and WI-3), a lower concentration of HPAIv in feces (WI-4), a higher survival of HPAIv during transport of aerosols (WI-7) and a lower ventilation rate in poultry houses based on values for broiler farms (WI-9) only had limited effects on the estimated risk.

## 4. Discussion

The estimated probability of an HPAI outbreak in a poultry house due to the wind-supported transmission of HPAIv via fecal particles from infected wild birds is very low, with a median value of 2.2 × 10^−3^ per bird-flu season. This is an overall estimate, averaged over different farm types, virus strains and wild bird species, and results indicate that uncertainty is relatively high. However, even under worst-case conditions, the probability is still low, with a 97.5 percentile value of 0.06, which equals an expected introduction to domestic poultry via this route once every 17 years.

Although the quantitative risk assessment model that we used is a stochastic risk model accounting for uncertainty and variability in input parameters, the model does not simulate infections in individual farms but rather calculates the probability of infections at the farm level and the sector level in the Netherlands. The estimated median daily exposure to HPAIv at the farm level is very low, with a value of 1.7 × 10^−7^ EID_50_, resulting in a low infection probability ($P_{inf,pf,d}$ = 7.5 × 10^−9^). If only considering days on which infection is possible, i.e., infected wild birds are present at the farm and weather conditions are suitable for the aerosolization of feces, the exposure is almost 10^4^ log_10_ higher at 1.4 × 10^−3^ EID_50,_ resulting also in an almost 10^4^ log_10_ higher infection probability ($P_{inf,pf,d}$ = 5.9 × 10^−5^).

Probability distributions of model input parameters represent both uncertainty and variability. We had, e.g., great uncertainty in the fraction of virus surviving during aerosolization and the dispersion of the virus before reaching the poultry house. Variability resulted from differences in HPAIv strains and wild bird species. Model parameters were estimated based on data for Anseriformes, mostly dabbling ducks, with data for mallards (*Anas platyrhynchos*) being most abundant. Also, model parameters were as much as possible based on values for HPAIv strains. However, if these were not available (e.g., for survival of virus), we used data for LPAI virus strains or influenza A viruses as the best alternative. Parameters on virus excretion in feces and the bird infectious dose were derived from studies in which values for multiple HPAIv strains were reviewed and compared [11,39,57,59]. High variations in these values were observed among HPAIv strains, making it difficult to decide on a representative value for this model.

Uncertain input parameters that had the greatest effect on model outcome were the fraction of virus retained after the dispersion of aerosols over a short distance ($F_{disp}$), the concentration of HPAIv in wild bird feces (${AI}_{fec\_conc}$), the number of wild birds at the farm yard ($N_{wb}$), the fraction of the day birds are present ($F_{pf}$) and the bird infectious dose (${BID}_{50}$) (Supplementary Table S2; Figure 3). The what-if analysis indicated that model results are also highly sensitive to assumptions on the survival of HPAIv during the drying of feces ($F_{surv\_dry}$) and the HPAI prevalence in wild birds (${Prev}_{wb}$) (Figure 4).

We had very limited data to estimate the fraction of virus retained after the dispersion of aerosols over a short distance ($F_{disp}$). There are multiple studies modeling the aerosol transmission of HPAI (e.g., [13,17,19,38,61]), but all these studies take an infected poultry farm as the source of infection rather than fecal droppings deposited by wild birds in the environment around a poultry farm. We expect a much lower probability of aerosol transmission via these fecal droppings for several reasons: the amount of virus excreted is less than that in an infected poultry flock, the environmental conditions (temperature, humidity) for aerosolization are less suitable outside the poultry house during the bird-flu season and the aerosols are emitted from ground level rather than from a ventilation outlet at 1.5 to 2 m height. Furthermore, it should be acknowledged that the processes of AI airborne transmission, such as aerosolization, transportation and deposition are complex and not fully understood, and all modeling studies need to make assumptions on these issues [38,62,63]. In our model, we have chosen a simple approach, where we estimated the remaining fraction after each step in which inactivation or loss of infection could occur rather than complex models accounting for, e.g., meteorological conditions.

The estimated values for virus shedding in feces (${AI}_{fec\_conc}$) were based on data from cloacal swabs, where we assumed that the concentration given in EID_50_/mL for cloacal swabs corresponds to the concentration in feces given as EID_50_/g [64]. We used data from a systematic literature review by Germeraad et al. [11] to estimate the concentration of HPAIv in wild bird feces. The estimates in this study were based on a meta-analysis of studies on HPAIv infections with both high-pathogenic and low-pathogenic AI viruses in multiple bird species. To estimate ${AI}_{fec\_conc}$, we only selected results for HPAI viruses (both H5 and H7 strains) in ducks. This estimate is therefore considered to cover the variation among virus strains and duck species. A recent study by Beerens et al. [50] confirmed that there is indeed variation in the amount of virus excreted in feces between wild bird species and HPAIv strains. Also, the amount of virus excretion is not stable over time, with virus titers decreasing after the first week of infection [58]. The distribution used for ${AI}_{fec\_conc}$ in the baseline scenario is likely to be an average over the full infectious period of the birds and does not account for peak titers, which have been observed to be between 4 and 6 log_10_ EID_50_/mL [50,58,60].

Very few data were available to estimate the number of ducks that are present on the farm yard ($N_{wb}$), i.e., at a short distance (<20 m) of the poultry houses. Few studies have actually closely monitored wild bird activity on poultry farms [2,20,27,28,29,30]. Predominantly song birds (order Passeriformes) are observed on the premises of poultry farms; members of the orders Anseriformes and Charadriiformes are hardly reported to visit areas close to poultry houses. Elbers and Gonzales [2,28] observed visits of wild fauna, including birds, to the free-range area of a layer farm in a high-risk area in the Netherlands. They concluded that dabbling ducks visited the outdoor facility only at night time in the period from November to May, i.e., especially during the bird-flu season, with most birds visiting in the months December, January and February. We used the observations by Elbers and Gonzales [2] to estimate the frequency of bird visits ($P_{wb}$), the number of birds visiting ($N_{wb}$) and the time spent on the farm yard ($F_{pf}$), as these were the only data available to quantify these parameters. These values are likely to be overestimates, as the outdoor facility was not paved and had water pools after rainy periods that might have attracted the wild birds. A study by Veen et al. [26] reported on the number of birds observed at a distance of <50 m from the farm buildings in different European countries based on limited observations. Only a few ducks were reported close to the farms (maximum of six over the total observation period), whereas other bird species were observed in much higher numbers. Similar observations were obtained by Elbers et al. [20] at a different layer and broiler farm in the Netherlands, despite the presence of waterways at a close distance to the farms in this study. Results of the sensitivity analysis clearly indicate that these parameters have high impact on model results (Supplementary Table S2; Figure 3).The estimated infection risk due to the wind-supported transmission of HPAIv to poultry farms via fecal particles from infected wild birds in the environment can thus be considered a worst-case estimate.

Studies on the infectious dose of HPAIv that has a 50% probability of infection in poultry, the ${BID}_{50}$, show that this value varies largely across HPAIv strains, poultry species and inoculation routes [39,57,59]. Sergeev et al. [39] compared the aerosol route of inoculation against other inoculation routes and concluded that the ${BID}_{50}$ is lowest for the aerosol route, with average values ranging from 0.8 to 1.2 EID_50_. Swaye and Slemons [59] reported ${BID}_{50}$ values for the intranasal route, which vary widely from 1.2 to 4.7 EID_50_, with an average value of 2.9 EID_50_. Accounting for a 30-fold lower effectivity of the intranasal route compared to the aerosol route [39], this average is only slightly higher than the values given by Sergeev et al. [39]. While one could hypothesize that the ${BID}_{50}$ in poultry is higher if the virus strain originates from ducks, this is not observed in the values reported in literature [25,39]. In the what-if analysis, we tried to account for this possibility and based our value for the ${BID}_{50}$ on an estimated ${BID}_{50}$ in chickens for a virus strain isolated from tufted ducks [25,60]. This resulted in a >100-fold lower risk of infection in poultry farms due to the wind-supported transmission of HPAIv derived from fecal particles from infected wild birds with an estimated median $P_{inf}$ of 1.4 × 10^−5^ (Figure 4).

Weather conditions during the Dutch bird-flu season allow for the prolonged persistence of HPAIv in feces [51,52]. However, the probability that the feces of wild birds will dry and aerosolize during the bird-flu season is likely to be low. Elbers [25] collected duck feces in the field and evaluated the meteorological conditions under which the drying of feces was observed (no precipitation and global sun irradiation ≥ 1000 J/cm^2^). We used these observations to estimate the probability that the feces of wild birds will aerosolize during the Dutch bird-flu season. Hardly any data were available to estimate the survival of HPAIv during this process of drying and aerosolization ($F_{surv\_dry}$). The few studies available indicated that the virus in feces is likely to be inactivated by drying within 1–2 days [34,51]. Experimental aerosolization studies of influenza virus reported varying levels of survival of the virus depending on the temperature and relative humidity applied during the experiments [64,65]. We considered, however, that aerosolization under experimental conditions is not likely to be representative of the drying process of feces under field conditions. In the baseline calculations, we used the observed decline in virus titer by Zarkov and Urumova [34] after one day of drying to parameterize $F_{surv\_dry}$. Considering that the drying of feces in the field is likely to take at least one week [25] and that Zarkov and Urumova [34] could no longer detect virus after two days of drying, this is likely to result in an overestimate of the infection risk for poultry farms.

Several studies were available to estimate the HPAI prevalence in wild birds (${Prev}_{wb}$) [22,23,24,41,42,66,67,68,69]. These studies varied with respect to the sampling method, sample size and matching of time and location with observed HPAI outbreaks in poultry farms. We decided to only include studies from Europe during the bird-flu season, as these were considered the most representative for our study. Extensive surveillance was performed during the bird-flu seasons of 2014/15 and 2016/17 in the Netherlands [22,23,24], indicating that the apparent prevalence in wild birds is low. We only included positive test results for HPAIv strains. Also, only samples of Anseriformes were taken as the denominator, as no HPAIv was detected in any other order of birds. Smaller sample sizes were taken during more recent studies in the UK and Italy [41,42]; these studies, however, indicated an almost 10-fold higher prevalence of HPAI in Anseriformes compared to the earlier studies. This might be the result of the changing HPAI situation in wild birds in recent years, where higher rates of morbidity and mortality have been observed, including in other orders such as Charadriiformes [70]. When analyzing each of the European studies separately, an increasing trend in apparent surveillance is indeed observed (Supplementary Figure S1). When including the higher prevalence rates based on the UK and Italian studies in the risk model (WI-1), the estimated probability of an infected poultry farm due to the wind-borne transmission of fecal particles from infected wild birds ($P_{inf}$) was increased 10-fold to a median value of 0.022 (95% uncertainty interval: 7.3 × 10^−4^ to 0.44) per bird-flu season (Figure 4), which equals an expected introduction in domestic poultry via this route once every 45 years, which is still very low. We did not account for the spatial clustering of infections in the model calculations, which might result in higher prevalence levels in wild birds in some areas and lower prevalence levels (or even absence of infection) in other areas. Although spatial clustering could result in a higher infection risk for individual farms, it will also result in lower risk levels for other farms. Using the overall prevalence level in wild birds in the model calculations thus resulted in an average infection risk for poultry farms in the Netherlands, leveling out the possible variation across individual farms. This will only have resulted in an underestimate of the risk if the spatial clustering of infections in wild birds coincides with the spatial clustering of poultry farms.

The input values used for this quantitative risk assessment were largely based on farming systems, wild bird behavior and environmental conditions observed in the Netherlands in the bird-flu season, which was defined as the period from October to April (European winter time). Caution is warranted when extrapolating results to other regions in the world or other seasons. For instance, Italy had a major HPAI outbreak in poultry during the 2017 summer period (July–November), and all primary outbreaks were attributed to indirect contact with wild birds [71]. It is unclear if the wind-borne transmission of aerosolized wild bird droppings could have played a role in this outbreak. Weather conditions during the Italian outbreak would anyway have been much more favorable for the aerosolization of fecal droppings than those during the Dutch bird-flu season. Also, the evolving epidemiology of HPAI with an increasing number of mammalian species reported to be infected could contribute to the contamination of the environment by secretions and excreta such as feces. However, no quantitative data are available (yet) to estimate the probability of the wind-supported transmission of HPAIv via aerosolized feces from infected mammals.

## 5. Concluding Remarks

Our model results indicate that the daily probability that the aerosolization of fecal droppings from wild birds in the vicinity of poultry farms would result in the infection of indoor-housed poultry in the Netherlands is extremely low. We estimated that this introduction route will result in an infected poultry farm during the Dutch bird-flu season once every 455 years (median value). Even under worst-case conditions (97.5 percentile value), this probability is still very low (once every 17 years). These results bring us to hypothesize that other risk factors, such as failures in strict and consistent compliance to biosecurity measures at the farm, might possibly be of more importance in HPAIv incursion on poultry farms [72,73,74]. Furthermore, this study provides guidance for the prevention of any possible wind-supported transmission of HPAIv to poultry farms via fecal particles from infected wild birds. The drying of HPAIv-contaminated fecal droppings from wild birds is a prerequisite for aerosolization, and this practically only happens during the bird-flu season when the droppings are deposited on concrete or stone-paved surfaces surrounding the premises. The probability of the occurrence of a chain of drying of HPAIv-contaminated wild bird feces, subsequent aerosolization and wind-borne transport of still-infectious HPAIv through air inlets of a poultry house is very low. To make this probability extremely low to negligible, it would be prudent for the poultry farmer to regularly check for the presence of wild bird droppings on the paved flooring around poultry houses and to safely remove these. This will also reduce the probability of the incidental introduction of HPAIv-contaminated wild bird droppings into the poultry house by sticking to the boots of people walking on the premises and entering poultry anterooms.

## Figures and Tables

**Table 1:** Parameters of the quantitative microbial risk assessment model.

Model Parameter	Description	Value	Source
$P_{wb}$	Daily probability that wild birds are present at the farm	Beta distribution with α = 43 and β = 95	[2]
$N_{wb}$	Number of wild birds at the farm yard on a day that birds are present	Pert distribution with min = 1, most likely = 6 and max = 24	[2]
WB	Expected number of wild birds at the farm yard	$P_{wb}\times N_{wb}$	Calculated parameter
$F_{pf}$	Fraction of the day that wild birds spend at the farm yard	Pert distribution with min = 3.4 × 10^−4^, most likely = 0.16 and max = 0.77	[2]
${Prev}_{wb}$	Apparent HPAI prevalence in wild birds	Beta distribution with α = 35 and β = 8521	[22,23,24]
${Fec}_{wb\_dry,d}$	Daily amount of feces excreted by wild birds (dry weight in grams)	Lognormal distribution with mean = 36.9 and SD = 4.37	[31]
$C_{dry\_wet}$	Conversion factor from dry to wet feces	5	[32]
${Fec}_{wb,d}$	Daily amount of feces excreted by wild birds (wet weight in grams)	$C_{dry\_wet}\times {Fec}_{wb\_dry,d}$	Calculated parameter
${AI}_{fec\_conc}$	Concentration of HPAIv in wild bird feces (log_10_ EID_50_/g)	Lognormal distribution with mean = 3.8, 2.5 percentile = 3.15 and 97.5 percentile = 4.5	[11]
$F_{weather}$	Fraction of days with suitable weather conditions for the aerosolization of feces	0.014	[33]
$F_{surv\_dry}$	Survival of HPAIv during the drying of feces	10^−3.25^	[34]
$F_{disp}$	Fraction of virus retained after the dispersion of aerosols over a short distance	Uniform distribution with min = −2 log_10_ and max = −0.5 log_10_	[35]
$F_{surv\_trans}$	Survival of HPAIv during air transport	Uniform distribution with min = 0.61 and max = 0.70	[36]
$F_{barn}$	Fraction of aerosols entering the barn	0.05	Estimate based on surface area of ventilation openings in poultry barns
VR	Ventilation rate of poultry house (layers) (m^3^/animal/hour)	Pert distribution with min = 1.1, most likely = 3.5 and max = 9	[37]
RV	Respiratory volume of chickens (layers) (m^3^/hour)	0.0224	[38]
$F_{inhale}$	Fraction of air in the poultry house that is inhaled by the animals	RV/VR	Calculated parameter
${BID}_{50}$	Bird infectious dose (log_10_ EID_50_)	Normal distribution with mean = 1.2 and SD = 0.2	[39]
DR	Dose–response parameter (EID_50_^−1^)	$\ln(2)/10^{{BID}_{50}}$	Calculated parameter
$N_{pf}$	Number of poultry farms in the Netherlands	1353	[40]
D	Length of bird-flu season (days)	212.25	October–April (number corrected for leap years)

**Table 2:** What-if scenarios explored with the quantitative microbial risk assessment model.

Scenario	Description	Model Parameter	Baseline Value	New Value	Source
WI-1	Higher prevalence in wild birds	${Prev}_{wb}$	Beta distribution with α = 35 and β = 8521	Beta distribution with α = 41 and β = 930	[41,42]
WI-2	Lower dry weight (g) of fecal droppings (data for adult ducks)	${Fec}_{wb,dry,d}$	Lognormal distribution with mean = 36.9 and SD = 4.37	Lognormal distribution with mean = 26.3 and SD = 11.5	[48]
WI-3	Higher dry weight (g) of fecal droppings (data for greylag geese)	${Fec}_{wb,dry,d}$	Lognormal distribution with mean = 36.9 and SD = 4.37	100	[32]
WI-4	Lower concentration of HPAIv in feces (log_10_ EID_50_/g) (data for H5N8-2014 in Eurasian wigeon)	${AI}_{fec\_conc}$	Lognormal distribution with mean = 3.8, 2.5 percentile = 3.15 and 97.5 percentile = 4.5	Lognormal distribution with mean = 3.38 and SD = 0.44	[50]
WI-5	Higher concentration of HPAIv in feces (log_10_ EID_50_/g) (data for H5N8-2016 in Eurasian wigeon)	${AI}_{fec\_conc}$	Lognormal distribution with mean = 3.8, 2.5 percentile = 3.15 and 97.5 percentile = 4.5	Lognormal distribution with mean = 4.96 and SD = 0.77	[50]
WI-6	Lower survival of HPAIv during the aerosolization (drying) of feces	$F_{surv\_dry}$	10^−3.25^	10^−6.5^	Estimate based on [34]
WI-7	Higher survival of HPAIv during transport of aerosols	$F_{surv\_trans}$	Uniform distribution with min = 0.61 and max = 0.70	0.99	[17]
WI-8	Higher fraction of virus retained after the dispersion of aerosols	$F_{disp}$	Uniform distribution with min = −2 log_10_ and max = −0.5 log_10_	Uniform distribution with min = 0.63 and max = 0.78	[53]
WI-9	Lower ventilation rate of poultry houses based on broilers (m^3^/animal/hour)	VR	Pert distribution with min = 1.1, most likely = 3.5 and max = 9	Pert distribution with min = 0.1, most likely = 2.1 and max = 9.6	[37]
WI-10	Higher bird infectious dose based on H5N8 virus isolated from a tufted duck (log_10_ EID_50_)	${BID}_{50}$	Normal distribution with mean = 1.2 and SD = 0.2	Pert distribution with most likely = 4.85, 2.5 percentile = 4.23 and 97.5 percentile = 5.51 MINUS 1.48 to correct for the aerosol inoculation route	[25,39,60]

## Data Availability

Data are contained within the article.

## Supplementary Materials

The following supporting information can be downloaded at: https://www.mdpi.com/article/10.3390/pathogens13070571/s1, Table S1: Average values of daily mean, minimum and maximum temperatures; daily precipitation; and daily global radiation per month in De Bilt, The Netherlands, during the period 1993–2022 and the average number of days per month that meet the criteria for the aerosolization of fecal droppings; Table S2: Correlation coefficients of uncertain input parameters with the overall probability of at least one infected poultry farm during the bird-flu season ($P_{inf}$); Figure S1: Apparent prevalence of HPAI in wild waterfowl (mean and 95% uncertainty interval).
