# Supplementary material for: Quantitative Risk Assessment of Wind-Supported Transmission of Highly Pathogenic Avian Influenza Virus to Dutch Poultry Farms via Fecal Particles from Infected Wild Birds in the Environment
Source: Pathogens. 2024 Jul 8;13(7):571. doi: 10.3390/pathogens13070571 (PMC11279698; doi:10.3390/pathogens13070571)
Supplement: Supplementary file 1 [file pathogens-13-00571-s001.zip › pathogens-3065873-supplementary.pdf]

Supplementary Materials

Weather conditions during Dutch bird-flu season

Daily data on weather conditions in the Netherlands were downloaded from KNMI - Daggegevens van het weer in Nederland for station "De Bilt", which is one of the main weather stations in the Netherland, is centrally located, and has the longest historical range of data. The KNMI database contains multiple parameters on temperature, rainfall, wind, sunshine, etc. Data of the period 1993-2022 were used to evaluate the conditions during the Dutch bird-flu season (October – April) (Table S1).

We assumed that aerosolization was only possible at days at which global radiation (Q in the database) was > 1000 J/cm^2 AND precipitation (RH in the database) was < 0.1 mm. We estimated the number of days in the months January, February, March, April, October, November, and December in this 30-year period that met these conditions AND that had at least 6 preceding days that also met the conditions, which was 89 in total. This number was then divided by the total number of days in the period evaluated (30×(31+28.25+31+30+31+30+31)=6367.5) to arrive at the probability of suitable weather conditions during the Dutch bird-flu season.

Table S1. Average values of daily mean, minimum and maximum temperatures; daily precipitation; and daily global radiation per month in De Bilt, The Netherlands, during the period 1993-2022 and the average number of days per month that meet the criteria for the aerosolization of fecal droppings.

Month	Mean temperature (degrees Celsius)	Minimum temperature (degrees Celsius)	Maximum temperature (degrees Celsius)	Precipitation (mm)	Global radiation (J/cm^2)	No. of days with rainfall < 0.1 mm	No. of days with global radiation > 1000 J/cm^2	No. of days at which aerosolization is possible[a]
January	3.7	1.0	6.2	2.3	233	12	0	0.00
February	4.1	1.0	7.3	2.4	463	11	1	0.00
March	6.4	2.2	10.5	1.8	882	14	11	0.63
April	9.8	4.5	14.8	1.4	1427	16	21	2.30
May	13.4	7.9	18.4	2.0	1745	15	24	2.20
June	16.3	10.9	21.2	2.3	1871	15	24	1.53
July	18.2	12.9	23.1	2.7	1789	15	25	2.27
August	17.9	12.5	22.9	2.7	1524	15	23	2.47
September	14.7	10.0	19.5	2.6	1060	14	15	0.73
October	11.1	7.2	15.1	2.6	609	14	3	0.03
November	7.1	4.0	10.0	2.6	286	10	0	0.00
December	4.3	1.6	6.7	2.7	175	11	0	0.00

[a] Days with global radiation > 1000 J/cm^2 AND precipitation < 0.1 mm AND at least 6 preceding days that also met these conditions.

Sensitivity analysis

Table S2. Correlation coefficients of uncertain input parameters with the overall probability of at least one infected poultry farm during the bird-flu season (P_{inf}).

Model parameter	Description	Correlation coefficient
F_{disp}	Fraction of virus retained after dispersion of the aerosols over a short distance	0.59
AI_{fec_conc}	Concentration of HPAIv in wild bird feces	0.45
F_{pf}	Fraction of the day wild birds spent at farm yard	0.40
N_{wb}	Number of wild birds at farm yard on a day that birds are present	0.31
BID_{50}	Bird infectious dose	-0.26
VR	Ventilation rate of poultry house	-0.22
$Prev_{wb}$	Apparent HPAI prevalence in wild birds	0.10

P_{wb}	Daily probability that wild birds are present at the farm	0.08
$Fec_{wb\_dry,d}$	Daily amount of feces (dry weight) excreted by wild birds	0.06
F_{surv_trans}	Survival of HPAIv during air transport	0.02

Apparent prevalence of HPAI in wild birds

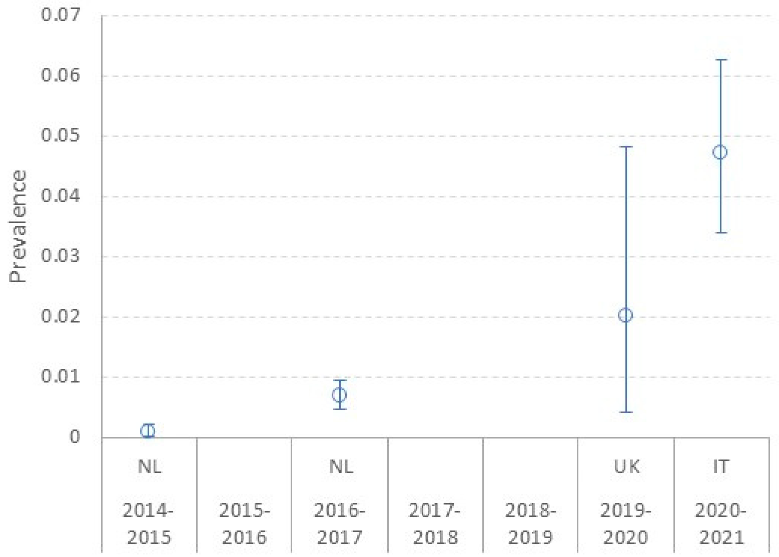

Figure S1. Apparent prevalence of HPAI in wild waterfowl (mean and 95% uncertainty interval). Source: Verhagen et al., 2015; Poen et al., 2016; Poen et al., 2018; Gobbo et al., 2021; Wade et al., 2022.

References

1. Gobbo, F.; Fornasiero, D.; De Marco, M.A.; Zecchin, B.; Mulatti, P.; Delogu, M.; Terregino, C. Active Surveillance for Highly Pathogenic Avian Influenza Viruses in Wintering Waterbirds in Northeast Italy, 2020-2021. *Microorganisms* **2021**, 9(11), 2188. doi:10.3390/microorganisms9112188.
2. Poen, M.J.; Verhagen, J.H.; Manvell, R.J.; Brown, I.; Bestebroer, T.M.; van der Vliet, S.; Vuong, O.; Scheuer, R.D.; van der Jeugd, H.P.; Nolet, B.A.; Kleyheeg, E.; Müskens, G.J.D.M.; Majoor, F.A.; Grund, C.; Fouchier, R.A.M. Lack of virological and serological evidence for continued circulation of highly pathogenic avian influenza H5N8 virus in wild birds in the Netherlands, 14 November 2014 to 31 January 2016. *Euro Surveill* **2016**, 21(38), 30349. doi:10.2807/1560-7917.ES.2016.21.38.30349.
3. Poen, M.J.; Bestebroer, T.M.; Vuong, O.; Scheuer, R.D.; van der Jeugd, H.P.; Kleyheeg, E.; Eggink, D.; Lexmond, P.; van den Brand, J.M.A.; Begeman, L.; van der Vliet, S.; Müskens, G.J.D.M.; Majoor, F.A.; Koopmans, M.P.G.; Kuiken, T.; Fouchier, R.A.M. Local amplification of highly pathogenic avian influenza H5N8 viruses in wild birds in the Netherlands, 2016 to 2017. *Euro Surveill* **2018**, 23(4), 17-00449. doi:10.2807/1560-7917.ES.2018.23.4.17-00449.
4. Verhagen, J.H.; van der Jeugd, H.P.; Nolet, B.A.; Slaterus, R.; Kharitonov, S.P.; de Vries, P.P.; Vuong, O.; Majoor, F.; Kuiken, T.; Fouchier, R.A. Wild bird surveillance around outbreaks of highly pathogenic avian influenza A(H5N8) virus in the Netherlands, 2014, within the context of global flyways. *Euro Surveill* **2015**, 20(12), 21069. doi:10.2807/1560-7917.ES2015.20.12.21069.
5. Wade, D.; Ashton-Butt, A.; Scott, G.; Reid, S.M.; Coward, V.; Hansen, R.D.E.; Banyard, A.C.; Ward, A.I. High pathogenicity avian influenza: targeted active surveillance of wild birds to enable early detection of emerging disease threats. *Epidemiol Infect* **2023**, 151, e15. doi:10.1017/S0950268822001856.
